# Practice of antimalarial prescription to patients with negative rapid test results and associated factors among health workers in Oyo State, Nigeria

**DOI:** 10.11604/pamj.2018.30.229.13231

**Published:** 2018-07-26

**Authors:** Akinfemi Oyewumi Akinyode, IkeOluwapo Oyeneye Ajayi, Muhammed Sani Ibrahim, Joshua Odunayo Akinyemi, Olufemi Olamide Ajumobi

**Affiliations:** 1 Nigeria Field Epidemiology and Laboratory Program, Abuja, Nigeria; 2 Department of Epidemiology and Medical Statistics, University of Ibadan, Nigeria; 3 Department of Community Medicine, Ahmadu Bello University, Zaria, Nigeria; 4 National Malaria Elimination Programme, Federal Ministry of Health, Abuja; 5 Nigeria, Africa Field Epidemiology Network, Abuja, Nigeria

**Keywords:** Rapid diagnostic tests, antimalarial, prescriptions, Nigeria

## Abstract

**Introduction:**

Contrary to World Health Organizations recommendations, health workers (HWs) still prescribe antimalarials to malaria rapid diagnostic test (mRDT)-negative patients, thus increasing overuse and the risk of parasite resistance to the antimalarials. The reasons for this are not clear. We identified factors associated with antimalarial prescription to mRDT-negative patients.

**Methods:**

We conducted a cross-sectional study among 423 HWs. Data on socio-demographic characteristics, training, supervision experience and fever management practices were collected. We tested associations between independent variables and prescription of antimalarials to mRDT-negative patients using Chi square and logistic regression at p < 0.05.

**Results:**

The HWs were mostly community health workers (58.6%), with mean age of 41.0 (±8.8) years and 13.6 (± 9.0) years of professional practice. Females were 322 (76.1%) and 368 (87%) were married. Of the 423 HWs interviewed, 329 (77.8%) received training on mRDT use, 329 (80.6%) received supervision and 129 (30.5%) had good knowledge of causes of fever. Overall, 110 (26.0%) of the HWs prescribed antimalarials to mRDT-negative patients. A higher proportion of non-trained vs trained HWs [Adjusted Odds Ratio (aOR) = 4.9; 95% Confidence Interval (CI) (2.5-8.3)], and HWs having poor knowledge vs HWs having good knowledge of causes of fever [aOR = 1.9; 95% CI (1.0-3.5)], prescribed antimalarials to mRDT-negative patients.

**Conclusion:**

HWs' lack of training on mRDT use and poor knowledge of causes of fever were associated with prescription of antimalarials to mRDT-negative patients. We recommend training on management of fever and mRDT use to reduce such inappropriate antimalarial prescriptions.

## Introduction

Nearly half of the world's population are at risk of malaria, with 91 countries and territories in the world still having ongoing transmission of the disease. Over 212 million cases and 429,000 deaths from malaria were reported in 2015, with 92% of the deaths occurring in Africa. Nigeria alone accounted for 26% of the global malaria deaths [1]. Mortality from malaria can be prevented if it is diagnosed early and effective treatment is commenced promptly [2, 3]. Microscopy is the gold standard for diagnosis of malaria. However, it is not feasible for use in all facilities because it requires the use of a functional microscope, regular supply of reagents, trained laboratory personnel and reliable supply of electricity, all of which are either not available at all or not guaranteed in many facilities in endemic countries [4, 5]. However, the development of the malaria rapid diagnostic test (mRDT) has increased the opportunity for parasitological diagnosis of malaria where microscopy is not available. They neither require the use of specialized equipment, specialized training nor electricity [6]. They have been proven to be able to reliably detect the presence or absence of malaria parasites from blood with sensitivity and specificity as high as 100% and 98.5% respectively [7]. However, despite its high sensitivity and specificity, health workers often disregard the results of mRDTs. As much as 72% of health workers prescribe antimalarial medicine to mRDT negative patients in some settings [8]. Prescribing antimalarial medicine to mRDT negative patients is in contradiction to the World Health Organisation's guidelines and Nigeria's National Malaria Treatment Policy which recommend that, where parasitological diagnosis is accessible, antimalarial medicine should only be prescribed to patients with positive parasitological test results [9, 10]. Prescribing antimalarial medicine to mRDT negative patients could result in over-prescription and waste of the antimalarial drugs, economic losses, mismanagement of fever cases and could contribute to resistance to the currently effective artemisinin based combination therapy used for treating malaria [11-15]. Rather than prescribing an antimalarial to a patient whose mRDT result is negative, it is recommended that other causes of fever should be explored [9, 10]. Expert microscopy remains an option if mRDT result is in doubt. Studies have been done to identify factors that influence prescription of antimalarial medicine to mRDT negative individuals. Factors such as perceptions about mRDTs by health workers and by clients, lack of skills and tools for differential diagnosis, fear of wrong diagnosis, peer influence, and patients' preferences have been identified to influence health workers' practice [16-18]. The role of training, supervision and use of job aids have also been reported [18]. Most of the studies conducted have been qualitative studies and the strength of influence of the identified factors on practice were not evaluated [16-21]. Evidence also suggests that the role of factors that influence practices of health workers are context specific [22]. In Oyo State, only few studies have been done to identify the practices of health workers relating to the use of mRDTs. Also, there are paucity of studies done to identify the reasons why health workers do not comply with the mRDT results. We conducted a quantitative survey to assess the treatment practices of health workers for mRDT-negative patients in Oyo State and to identify the factors associated with prescribing antimalarial medicine to such patients.

## Methods

**Study area:** The study was conducted among health workers in Oyo State from June to August 2016. Oyo State is in the South-west geo-political zone of Nigeria. It is situated within longitude 3.94° East of the Meridian Longitude Zero and Latitude 7.84° North of the Equator. The climate in Oyo state is characterised by rainfalls as high as 190 mm in the peak months of the rainy seasons and high humidity sometimes as much as 86%. Atmospheric temperature in the state often ranges from 24.1°C to 28.7°C [23]. Transmission of malaria in the State is perennial. There are 1,729 registered health facilities across the 33 local government areas (LGAs) of the State. Seven hundred and sixty-one of the facilities are public health facilities while 961 are privately owned. The public health facilities consist of two tertiary, 43 secondary, and 716 primary health facilities. All the 1,729 facilities offer preventive and curative services for malaria. Such services include, routine provision of long lasting insecticidal nets to children and pregnant women, intermittent preventive treatment of pregnant women for malaria using Sulphadoxine-Pyrimethamine (SP), and treatment of uncomplicated malaria using Artemisinin Combination Therapy (ACT). Cases of severe malaria are managed only in secondary and tertiary facilities. Seven hundred and twenty-three (41.8%) of the facilities were benefiting from the routine distribution of the mRDTs. Of the 723 facilities, 602 (83.3%) were public primary facilties, 27 (3.7%) were public secondary facilities while 94 (13.0%) were private facilities. Malaria treatment and prevention services are provided to patients free of charge in public health facilities and at subsidized rates in private facilities. Government, development partners and non-governmental organizations fund the services.

**Study design and population:** A cross sectional study was conducted among health workers in private and public facilities in Oyo State. Health workers who worked in health facilities within Oyo State were selected to participate in the study. Those who worked in health facilities which received routine supply of mRDT kits and whose job description in the facility included diagnosis and treatment of malaria were considered. Among these health workers, those that were not available at the time of the study were excluded.

**Sample size estimation:** A minimum sample size of 381 was calculated the required sample size using the formula for cross-sectional studies.

$$n=\frac{1.96^{2}\times p(1-p)}{d^{2}}$$

Where the proportion of health workers prescribing antimalaria to mRDT negative patient of *(p)* of 45.5% [24] and a level of precision *(d)* of 5%.

**Sampling procedure:** We selected the health workers using a multistage sampling technique. In the first stage, two thirds (22) of the LGAs in Oyo state were selected by simple random sampling. In the second stage, from the list of the 497 health facilities that received routine supply of mRDT kits in the selected LGAs, one third (166) were selected by simple random sampling. In the third stage, we selected health workers from the selected facilities by proportional allocation. The sample size was allotted to each of the facilities proportionately to the number of the health workers in the facility. Health workers were then selected from the list of the health workers in each facility by balloting to fill the quota allotted to the facility.

**Data collection:** A questionnaire was design using questions adapted from the study instruments of previous study in Uganda [16]. The questionnaire was face validated by experts for correctness and suitability to address the objectives of the study. The questionnaire was pretested among health workers in an LGA that was not selected for the study. The questionnaires were administered by 22 interviewers who were trained at a 2-day workshop. The interview was conducted in secluded area of the clinic.

**Data analysis:** The main outcome of this study was prescribing antimalarial medicine to mRDT negative patients. The independent variables included age, sex, marital status, professional cadre, duration of professional practice of health worker, ownership of health facility where health worker practiced, level of care of the facility, location of health facility, training on mRDT use, supportive supervision and health workers knowledge of causes of fever. Knowledge of fever was assessed by asking patients to mention four causes of fever apart from malaria. Illnesses which had fever as a symptom or complication, according to the guidelines on integration management of childhood illnesses, were scored as correct answers to the question. Health workers were required to mention 4 causes of fever. Health workers who mentioned 3 or 4 causes of fever correctly were classified as having good knowledge of causes of fever. Those who mentioned none, 1 or 2 correctly were classified as having poor knowledge of causes of fever. Data was summarised using descriptive statistics such as means and standard deviation and proportions. Association between prescription of antimalarial medicine to mRDT negative patients and each independent variable was assessed using Chi square test at 5% level of significance and predictors of antimalarial use in mRDT negative patients were ascertained at logistic regression.

**Ethical approval:** Ethical approval was obtained from the Ethical Review Committee of Oyo State Ministry of Health before commencement of the study (References number: - AD 13/471/1058, date: - 15^th^ of March 2016). Permission was obtained from heads of health facilities, and informed consent was obtained from health workers before interviewing them. Confidentiality of the interview was maintained.

## Results

Overall, 423 health workers were interviewed. Their mean (SD) age was 41(9) years. They were mostly females (76.1%), married (87.0%), Community Health Workers (75.2%) among which 105 (24.8%) have been in practice for more than two decades. Majority, (75.2%) worked in public, (82.0%) in primary and urban (53.4%) health facilities respectively. Three hundred and twenty-two (77.8%) had received training on the use of RDT for case management of malaria, 129 (30.5%) had good knowledge of causes of fever, and 329 (80.6%) had received supportive supervision (Table 1) When a patient's malaria RDT result was negative, 141 (24.3%) of the health workers prescribed antimalarial drugs to the patients. The age of health workers, marital status, duration of professional practice, ownership of health facility where health workers practice, level of care of facility, location of facility, training on use of RDT in malaria case management, health worker's knowledge of causes of fever and health workers experience with supportive supervision showed statistically significant association with prescription of antimalarial drugs to malaria RDT negative patients (Table 2). After controlling for confounders, only lack of previous training on mRDT use and poor knowledge of causes of fever were independently associated with prescription of antimalarial medicine to mRDT negative patients. (Table 3).

**Table 1:** Characteristics of respondents (N = 423)

Characteristics	Frequency	Percentage
**Age Group (Years)**		
20 to 29	46	10.9
30 to 39	122	28.8
40 to 49	177	41.8
50 and above	78	18.5
**Sex**		
Male	101	23.9
Female	322	76.1
**Marital Status**		
Married	368	87.0
Not married	55	13.0
**Professional Cadre**		
Community Health Worker	248	58.6
Nurse	99	23.4
Medical Assistant	45	10.6
Medical Laboratory Scientist/Technician	17	4.0
Doctor	14	3.3
**Duration of Clinical Practice**		
0 – 4 years	83	19.6
5 – 9 years	71	16.8
10 – 14 years	97	22.9
15 – 19 years	67	15.8
≥ 20 years	105	24.8
**Ownership of facility of practice**		
Private	105	24.8
Public	318	75.2
**Level of Care of facility of practice**		
Primary	347	82.0
Secondary	76	18.0
**Location of Facility of practice**		
Rural	197	46.6
Urban	226	53.4
**Training on Use of RDT**		
Trained	329	77.8
Not trained	74	22.2
Supportive Supervision *		
Did not receive supervision	79	19.4
Received supervision	329	80.6
**Knowledge of causes of fever**		
Poor knowledge	294	69.5
Good knowledge	129	30.5

* Here, N = 408

**Table 2:** Factors associated with prescription of antimalarial medicine to RDT negative patients

Characteristics	Prescribes antimalarial medicine n (%)	Does not prescribe antimalarial medicine n (%)	Total n (%)	X^2^	P Value
**Age Group (Years)**					
20 to 29	23 (50)	23 (50)	46 (10.9)		
30 to 39	34 (27.9)	88 (72.1)	122 (28.8)		
40 to 49	32 (18.1)	145 (81.9)	177 (41.8)	23.67	< 0.0001
50 and above	13 (16.7)	65 (83.3)	78 (18.5)		
**Sex**					
Male	23 (22.8)	78 (77.2)	101 (23.9)	0.13	0.718
Female	79 (24.5)	243 (75.5)	322 (76.1)		
Marital Status					
Married	78 (21.2)	290 (78.8)	368 (87.0)	13.17	0.0003
Not Married	24 (43.6)	31 (56.4)	55 (13.0)		
**Professional Cadre**					
Community Health Worker	53 (21.3)	195 (78.6)	248 (58.6)		
Nurse	23 (23.2)	76 (76.8)	99 (23.4)		
Medical Assistant	14 (31.1)	31 (68.9)	45 (10.6)	10.28	0.068
MLS/T	8 (47.1)	9 (52.9)	17 (4.0)		
Doctor	4 (28.6)	10 (71.4)	14 (3.3)		
**Duration of Practice**					
Less than 5 years	29 (34.9)	54 (65.1)	83 (19.6)		
5 – 9 years	18 (25.3)	53 (76.7)	71 (16.8)		
10 – 14 years	26 (26.8)	71 (73.2)	97 (22.9)	10.77	0.029
15 – 19 years	12 (17.9)	55 (82.9)	67 (15.8)		
≥ 20 years	17 (16.2)	58 (83.8)	105 (24.8)		
**Ownership of HFP**					
Private	36 (33.4)	69 (65.7)	105 (24.8)	11.66	0.005
Public	66 (20.8)	252 (79.2)	318 (75.2)		
**Level of Care of HFP**					
Secondary	30 (39.5)	46 (60.5)	76 (18.0)	11.94	< 0.0001
Primary	72 (20.7)	275 (79.3)	347 (82.0)		
Location of HFP					
Urban	69 (30.5)	157 (69.5)	226 (53.4)	10.92	< 0.0001
Rural	33 (16.8)	164 (83.2)	197 (46.6)		
Training on use of RDT					
Not trained	50 (53.2)	44 (46.8)	94 (22.2)	55.84	< 0.0001
Trained	52 (15.8)	277 (84.2)	329 (77.8)		
**Supportive Supervision**					
Received no supervision	29 (36.7)	50 (63.3)	79 (19.4)	8.63	0.003
Received supervision	69 (21.0)	260 (79.0)	329 (80.6)		
Knowledge of CF					
Poor knowledge	79 (26.9)	215 (73.1)	294 (69.5)	4.00	0.045
Good knowledge	23 (17.8)	106 (82.2)	129 (30.5)		

MLS/T: - Medical Laboratory Scientist/Technologist

HFP: - Health Facility of practice

CF: - Causes of fever in RDT negative patients

**Table 3:** Predictors of health workers’ prescription of antimalarial medicine to RDT negative patients

Characteristics	Adjusted Odds Ratio	95% Confidence Interval for Adjusted Odds Ratio	P -Value
**Age Group (Years)**			
20 to 29	1		
30 to 39	0.72	0.27 – 1.95	
40 to 49	0.53	0.19 – 1.51	0.646
50 and above	0.57	0.16 – 1.97	
**Marital Status**			
Married	1		
Not married	1.44	0.59 – 3.53	0.424
**Duration of Practice**			
Less than 5 years	1		
5 – 9 years	0.66	0.27 – 1.61	
10 – 14 years	1.68	0.74 – 3.82	0.221
15 – 19 years	0.83	0.32 - 317	
≥ 20 years	0.94	0.37 – 2.40	
**Ownership Status of HFP**			
Public	1		
Private	1.41	0.58 -3.41	0.448
**Level of Care of HFP**			
Primary	1		
Secondary	1.67	0.68 – 4.14	0.274
**Location of HFP**			
Rural	1		
Urban	1.60	0.93 – 2.75	0.092
**Training on the use RDT**			
Trained	1		
Not Trained	4.59	2.53 – 8.29	< 0.0001
**Supportive Supervision**			
Received Supervision	1		
Did Not receive Supervision	1.50	0.80 – 2.82	0.424
**Knowledge of causes of fever**			
Good knowledge	1		
Poor Knowledge	1.89	1.04 – 3.45	0.037

HFP – Health facility of practice

1 – Reference value

## Discussion

In this study, a relatively high proportion of health workers were found to prescribe antimalarial medicine to mRDT negative patients. Lack of training on mRDT use and Poor knowledge of causes of fever among health workers were independently associated with prescribing antimalarial drugs to mRDT negative patients. High proportions of health workers prescribing antimalarial medicine tor mRDT negative patients has health and economic implications. It could result in wastage of the antimalarial drugs, increased cost of treatment, increased morbidity and mortality from non-malaria fevers and increased risk of resistance to the currently effective antimalarial drugs [11-15]. Contrary to our study which focused on proportion of health workers prescribing antimalarial medicine to mRDT negative patients, most studies focused on proportion of mRDT negative patients receiving antimalarial prescriptions. Although proportion of patients receiving inappropriate prescriptions does not necessarily reveal proportion of health workers offering inappropriate prescriptions, it could give an idea of the proportion of health workers offering such prescriptions. Using proportion of mRDT patients receiving antimalarial prescription as proxy for proportion of health workers prescribing antimalarial medicine to mRDT negative patients, our finding of a relatively high proportion of health workers prescribing antimalarial medicine to mRDT negative patients is consistent with findings from previous studies in Nigeria. In a country-wide study by Mokuolu et al in 2016, Health workers were found to have prescribed antimalarial drugs to 26% of mRDT negative patients [25], although the above study was conducted in private facilties compared with ours that was done in both private and public. Similar findings have also reported in other countries. A prevalence close to our finding (22% Vs 24%) was obtained from a meta-analysis of 14 studies across 9 countries in sub-Saharan Africa [26]. However, lower prevalence was reported in some other studies. In a study in Uganda, only 4% of mRDT negative patients received antimalarial medicine [16]. In this study, there had been recent trainings on mRDT use and supervision to selected sites. The practice of prescribing antimalarial medicine to mRDT negative patients was relatively higher in some other studies compared with ours. In a certain study in Malawi, 58% of mRDT negative patients received antimalarial prescription [27].

In another study carried out in Tanzania, 72% of mRDT negative patients were found to receive antimalarial drugs [8]. It is speculated that health workers probably had more confidence in the mRDTs with the passage of time as these studies were conducted in 2009 and 2011 respectively. Lack of training of health workers on mRDT use was strongly associated with prescribing antimalarial medicine to mRDT negative patients. Majority of health workers who were not trained, prescribed the medicines to mRDT negative patient. Findings from previous studies showed that training on mRDT use improve compliance with mRDT results. Prescription of antimalarial medicine was low in settings where there had been training of health workers prior to the study, as reported above for the study in Uganda [16]. A certain study in Zambia where health workers were trained on mRDT use before commencement of the study, revealed over 99% compliance with negative mRDT result [28]. In this study, health workers were more likely to prescribe antimalarial medicine to mRDT negative patients if they had poor knowledge of causes of fever. As found in previous studies, health workers appear to withhold antimalarial drugs from mRDT negative patients according to extent of their knowledge about differential diagnosis of malaria. They consider treating for other causes fever only if they knew the causes. In a qualitative study that was done in Uganda health workers were reportedly forced to prescribe antimalarial drugs to patients when they did not know what else could account for fever after exhausting the list of the differential diagnosis which they knew [17]. Findings from another study in Uganda also showed that limited ability to identify alternative causes of fever was one of the identified drivers of antimalarial prescription to mRDT negative patients [16]. In view of the finding that lack of knowledge is associated with poor adherence to negative mRDT results, it is logical to suggest that developing and training health workers with modules on management of alternative causes of fever in mRDT negative patients could help reduce inappropriate antimalarial prescription to the patients. Such modules should contain clear instructions on clinical actions to take when managing mRDT negative patients and enumerate common alternative causes of fever and their management. In addition to the treatment modules, algorithms may be developed and displayed in a conspicuous place in the health facilities to guide health workers when treating patients with negative mRDT results. Such algorithms may offer options on actions and investigations to be carried out when a patient's mRDT result is negative. Such algorithm may algorithms could be helpful to health workers when confronted with negative mRDT results.

**Limitations of the study:** Data collected were self-reported and were not independently verified. Some health workers may have given acceptable answers that are ideal, rather than what they do. To limit such wrong answers, we carefully framed the questions in the questionnaire to be able to obtain true information and we maintained confidentiality.

## Conclusion

Although over 75% of the health workers in this study withheld antimalarial medicine from mRDT negative patients, others were still prescribing antimalarial medicine to mRDT negative patients. Lack of training on the use of mRDTs and poor knowledge about possible causes of fever in a patient with negative mRDT result appear to be major factors determining the prescription of antimalarial medicine mRDT negative patients. To reduce to increase knowledge of health workers and to reduce this inappropriate prescription, we recommend regular training of health workers on case management of malaria. Trainings should also be done on identification and management of other causes of fever.

### What is known about this topic

- Health workers and patients' perceptions influence treatment decisions;
- System constraints such as poor referral system, lack of skills and tools for diagnosis is associated with inappropriate antimalarial prescriptions;
- Training, supervision influence prescription practices.

### What this study adds

- Training, supervision influence prescription practices.

## Competing interests

The author declare no competing interest.
